# The Effect of Internship Length on Self‐Efficacy and Clinical Competence: Accelerating Entry Into the Nursing Workforce in Saudi Arabia

**DOI:** 10.1155/jonm/6689249

**Published:** 2025-12-26

**Authors:** Sitah S. Alshutwi

**Affiliations:** ^1^ College of Nursing, King Saud Bin Abdulaziz University for Health Sciences, Riyadh, Saudi Arabia, ksau-hs.edu.sa; ^2^ King Abdullah International Medical Research Center (KAIMRC), Riyadh, Saudi Arabia, kaimrc.med.sa; ^3^ The Ministry of the National Guard, Riyadh, Saudi Arabia

**Keywords:** 1-year internship training, clinical competence, nursing intern, Saudi Arabia, self-efficacy

## Abstract

**Background:**

Limited studies have evaluated the impact of the length of internship training in relation to self‐efficacy and clinical competence and the factors influencing these variables among nurse interns, particularly in Saudi Arabia.

**Aim:**

The threefold aim of this study was to (1) determine the self‐efficacy and clinical competence of Saudi nursing interns, (2) assess the differences in their perceptions, and (3) evaluate the differences within the 1‐year internship program in the self‐efficacy and clinical competence of Saudi nursing interns.

**Design:**

The quantitative study employed a cross‐sectional design and comparative and predictive research approaches.

**Methods:**

A total convenience sample of 206 Saudi nursing interns participated in the study. T‐test and analysis of variance (ANOVA) were used to determine the differences in nursing interns’ perceptions. A multiple regression analysis was conducted to examine the extent to which various predictor variables accounted for variance in clinical competence among interns.

**Results/Findings:**

The nurse interns’ mean scores were 3.33/4 for self‐efficacy and 4.42/5 for clinical competence. No significant correlation was found between demographics and either variable. However, self‐efficacy correlated positively with clinical competence (*r* = 0.67,*p* < 0.001). A between‐group ANOVA showed significant differences in self‐efficacy (*F*(2, 203) = 19.00, *p* < 0.001) and clinical competence (*F*(2, 203) = 38.00, *p* < 0.001) across internship durations. Post hoc tests revealed significant increases in the 4‐ to 8‐month groups, with no significant difference between 8‐ and 12‐month groups.

**Implication:**

Given that no significant improvements were observed beyond the 8‐month mark, it is recommended that internship programs consider reducing the overall duration to 8 months. This would preserve educational effectiveness while optimizing the use of institutional and human resources. More importantly, such a change could help address the persistent nursing shortage by accelerating the transition of qualified nursing graduates into the workforce by enabling earlier entry into professional roles without compromising competence or confidence.

**Conclusion:**

In conclusion, the present study has strengthened the relationship between self‐efficacy and clinical competence. Higher self‐efficacy corresponds to an increase in clinical competence. Additionally, the findings suggest that the duration of the internship program is important for enhancing the clinical skills of nursing interns; however, no significant differences were found between interns at the 8‐ and 12‐month points in the program.

## 1. Introduction

Despite significant educational advancements and a rigorous nursing curriculum, Saudi Arabia continues to experience a shortage of nurses. According to the World Health Orgnization [1], there are only 5.6 nurses and midwives per 1000 people, highlighting a supply gap. This shortage is largely due to the limited number of nursing graduates entering the workforce [2].

Variances in the nursing curriculum requirements and required years of completion are being practiced in different countries. In Saudi Arabia, nurses must complete a 4‐year coursework plus a 1‐year internship program to earn the degree of Bachelor of Science in Nursing [3]. A comprehensive clinical training is a concrete foundation for newly graduated nurses. The adequacy of their clinical training will play a major role as they undergo transition from college education to clinical practice [4]. Thus, it is imperative that such transition must be met with scrutiny to ensure adequate clinical experiences are provided by academic institutions [5]. Moreover, the internship year is a requirement of the Saudi Commission for Health Specialties (SCFHS), Saudi Arabia’s regulatory body for professionals, as a part of their requirement for graduating nurses to register and take the licensure examination [6].

A number of studies have revealed that nursing graduates face challenges in role transition due to limited experience in clinical practice and other environmental factors, reflecting the complexity of the clinical settings and healthcare system [7, 8]. Ensuring that nursing students are adequately prepared and competent as registered nurses upon graduation is crucial. Although clinical practice is an integral component of nursing education, ensuring an adequate degree of preparedness among newly graduated nurses is becoming increasingly difficult [9].

Internship programs provide a practice‐based learning environment, allowing novice nurses to have a smooth transition from theoretical knowledge to real‐patient skill application. The aim of the 1‐year internship is to enhance the clinical competencies of nursing students through direct involvement in patient care under preceptor supervision [10]. A study conducted by van Rooyen et al. [11] revealed that a well‐planned program designed specifically for the final year students and new graduates enhanced the transition to professional nurses.

The effectiveness of an internship year for nursing students has been found valuable in determining clinical competence [3]. Furthermore, the effectiveness of the preceptorship during the internship year was perceived by students as highly effective and has been linked to clinical competence [12]. Nurse interns in the Al Jouf Region who participated in a study conducted by Alruwaili et al. [13] exhibited moderate to high levels of readiness for clinical practice during their internship year. Similarly, the work readiness among newly graduated nurses in Saudi Arabia is relatively high according to a study by Almotairy et al. [14].

Therefore, a proper transitional program with the guidance of the preceptors that helps nursing graduates foster their competence and self‐efficacy levels is essential to ensure performing safe, efficient, and effective nursing care compatible with high standards [12, 15]. More so, it is imperative to state that clinical competence and self‐efficacy are huge factors that ensure the delivery of high‐quality care among nursing students. Nursing competence is defined as the capacity of nurses to fulfil their required roles [16]. Self‐efficacy is the student’s ability to manage and perform efficiently in difficult, demanding situations [17].

Several studies revealed the correlation between the level of competence and self‐efficacy among nurses, indicating that self‐efficacy is a good indicator of excellent student clinical performance [18]. Most of the studies have revealed a positive linear correlation between clinical competence and self‐efficacy, indicating that a more confident student leads to a higher clinical competence level [15, 19]. Self‐efficacy is not only linked to nurses’ performance and competence levels, but it can also affect their emotional status and might lead to depression, anxiety, stress, and psychosis [20]. In contrast, other studies have found no correlation between clinical competence and student self‐efficacy [21].

Limited studies have evaluated the impact of internship on competence level and self‐efficacy among nursing graduates. A recent study evaluated a specific competence, including needlestick and sharp injury safety issues, and found a significant improvement in interns’ knowledge and practice regarding needlestick and sharp injury safety measures after implementation of training sessions [22]. Another study conducted in Saudi Arabia involving 92 undergraduate students found that internship programs showed improvement, with the availability, approachable attitude, and trustworthiness of preceptors identified as key factors positively influencing the interns’ clinical competence. [12]. Furthermore, a study stated that transition programs given to newly graduated nurses vary in length; some take up to a year to complete, while others are only 12–16 weeks in length [23]. Nevertheless, according to the National Council of State Boards of Nursing [24], healthcare institutions with such Transition to Practice programs (TTP) reported a decrease in attrition and improved patient outcomes. However, no clear measure of the effect of the length of the internship program on nursing interns’ self‐efficacy and competence has been established.

## 2. Aim

The aims of this study were to (1) determine the perceived self‐efficacy and clinical competence of Saudi nursing interns, (2) assess the differences in their perceptions, and (3) evaluate the differences within the 1‐year internship program in the self‐efficacy and clinical competence of Saudi nursing interns.

## 3. Methods

This study is reported in accordance with the Strengthening the Reporting of Observational Studies in Epidemiology (STROBE) guidelines for cross‐sectional/observational studies [25]. A completed STROBE checklist is provided as a supplementary file to ensure transparency and rigor in reporting.

### 3.1. Study Design

This study employed a cross‐sectional design, in which different cohorts of participants were surveyed at the 4‐, 8‐, and 12‐month stages of their internship. Rather than following the same individuals over time, separate groups were recruited at each interval. This approach was adopted due to the rolling nature of the internship program and to avoid challenges associated with participant attrition and maintaining longitudinal contact. While a longitudinal design offers the advantage of tracking individual‐level change over time, it was not adopted due to the high risk of attrition inherent in long‐term follow‐up studies. Attrition can introduce systematic bias if dropouts differ meaningfully from those who remain, thereby compromising the validity and generalizability of findings. Given the anticipated mobility and engagement challenges within our target population, a repeated cross‐sectional design was selected to minimize potential bias associated with differential loss to follow‐up. Surveying distinct cohorts currently at each internship stage allowed for timely and context‐specific data collection, offering insight into the experiences and perceptions relevant to each internship milestone. While this method does not permit within‐subject comparisons across time points, it provides a practical and valid framework for examining developmental trends during the internship period.

### 3.2. Settings and Sampling

The study was conducted at the College of Nursing, where nursing interns who graduated from the 4‐year baccalaureate nursing program completed a 1‐year internship at a single medical city comprising three tertiary and specialized hospitals. Convenience sampling method was selected due to practical constraints such as time, resource limitations, and accessibility to the target population. Specifically, the initial goal of this study was to obtain preliminary insights within a limited timeframe, and convenience sampling allowed for efficient and feasible data collection under these conditions. A sample of 206 nursing interns enrolled in the 1‐year internship training program. Sample size calculation software (Raosoft) was used to determine the sample size. The estimated target sample size was 159 nursing interns with a confidence interval of 95% and a margin of error of 0.05.

Nursing interns were included if they (1) were Saudis, (2) graduated from the same 4‐year baccalaureate nursing program, (3) officially enrolled in the 1‐year internship training program, (4) undertook their training at the same medical city, (5) were available during the data collection, and (6) willingly volunteered to participate in this study. Saudi nursing interns who did not graduate from the same program and those on long vacation leaves during the internship training program (e.g., maternity leave and deferment) were excluded from the study.

### 3.3. Instruments

The surveys were completed by the respondents at an average of 20 min and consisted of three parts. Part 1 collected nursing interns’ sociodemographic data, including social status, having relatives/friends studying/working in nursing, batch number (first = 1–4 months, second = 5–8 months, and third = 9–12 months) as length of internship, grade point average (GPA) score, and having an English proficiency test score.

Part 2 utilized the generalized self‐efficacy scale developed by Schwarzer and Jerusalem (1995)​ and measured the self‐efficacy of the interns. The scale consisted of ten statements that assessed the strength of nursing interns’ ability to respond to difficult or novel situations and deal with any associated obstacles or setbacks during their internship training program. This instrument used a four‐point Likert scale, ranging from 1 (*not at all true*), 2 (*barely true*), 3 (*moderately true*), and 4 (*exactly true*). The scores for each statement were summed up to obtain a total generalized self‐efficacy score. The higher the score, the greater the nursing interns’ generalized sense of self‐efficacy. The scale developers reported a high internal consistency, with Cronbach’s alphas ranging from 0.82 to 0.93 (Schwarzer & Jerusalem, 1995). Part 3 utilized the Clinical Competence Questionnaire (CCQ) developed by Liou and Cheng (2014)​ to measure the perceived clinical competence of nursing interns. The CCQ consisted of 47 competencies in four competency components with corresponding and specific competencies, as follows: (a) nursing professional behaviors (16 competencies), (b) general nursing skills (13 competencies), (c) core nursing skills (12 competencies), and (d) advanced nursing skills (six competencies). This scale was necessary because it gave nursing educators in nursing schools and hospitals a preliminary means of understanding nursing interns’ confidence in their clinical performance. The instrument used a five‐point Likert scale, with item response scores ranging from 1, “*do not have a clue*,” 2 “*know in theory, but not confident at all in practice*,” 3 “*Know in theory, can perform some parts in practice independently, and needs supervision to be readily available*,” 4 “*know in theory, competent in practice, need contactable sources of supervision*,” and 5 “*know in theory, competent in practice without any supervision*.” The total scores ranged from 46 to 230, with a higher score indicating nursing interns’ self‐perception of a higher level of clinical competence during their internship training program. The developers reported a Cronbach’s alpha of 0.98 for the entire CCQ (Liou & Cheng, 2014).

### 3.4. Data Collection Procedure

Data were collected over a period of 6 months. Participants were recruited using a convenience sampling method from one clinical site, a tertiary hospital. After obtaining informed consent, participants were invited to complete a structured questionnaire. The survey was administered through email invitations with a survey link. Participants were informed that their responses would remain anonymous and confidential, and they had the right to withdraw from the study at any time without penalty. All nursing interns were assured that their opinions and participation were confidential and would not affect their internship training program evaluation. To ensure data quality, only fully completed questionnaires were included in the final analysis. Ethical approval for the study was obtained from King Abdullah International Medical Research Center (Ref. no. IRB/0342/25). Privacy and confidentiality were also assured; no identifiers were collected, and all hard and soft copies of the data were kept in a secured place with access restricted to the research team only.

### 3.5. Data Analysis

Data were entered and analyzed using IBM‐SPSS Statistics Version 23.0. Prior to analysis, the dataset was screened for accuracy, outliers, and missing data. No missing data were found. Descriptive statistics were used to describe sociodemographic characteristics of nursing interns, such as social status, having relatives/friends studying/working in nursing, duration of internship (at 4, 8 and 12 months), GPA score, and having an English proficiency test score, and measure the levels of their perceptions regarding their self‐efficacy and clinical competence during the internship training program. T‐test and analysis of variance (ANOVA) were used to determine whether significant differences in nursing interns’ perceptions can be observed when grouped according to their sociodemographic characteristics. Lastly, sociodemographic characteristics and self‐efficacy were included as predictor variables in the model developed for nursing interns.

## 4. Results

### 4.1. Demographic Profile of Nursing Interns

Among the 206 nursing interns (response rate: 87.0%; Table 1), most were single (90.8%) and had relatives or friends studying or working in the nursing field (61.7%). Most respondents belonged to the first batch (1–4 months) of the internship program and had GPA scores between 3.61 and 4.00. Most nursing interns had taken a proficiency test (77.7%; *n* = 160), and of the 160, nearly half (48.8%) had proficiency test scores between 81 and 90.

**Table 1:** Demographic profile of nursing interns (*n* = 206).

Demographic profile	*f*	%
Social status		
Single	187	90.8
Married with no kids	11	5.3
Married with kids	8	3.9
Having relatives/friends studying/working in nursing		
Yes	127	61.7
No	79	38.3
Batch number		
First	92	44.7
Second	35	17.0
Third	79	38.3
GPA score		
2.51–3.00	17	8.3
3.01–3.50	56	27.2
3.61–4.00	73	35.4
4.01–4.50	51	24.8
4.51–5.00	9	4.4
Having proficiency test		
Yes	160	77.7
No	46	22.3
Proficiency test score (*n* = 160)		
Below 61	4	2.5
61–70	12	7.5
71–80	48	30.0
81–90	78	48.8
91–100	18	11.3

*Note: f* = frequency; % = percentage.

Abbreviation: GPA = grade point average.

Both scales used in this study revealed an excellent reliability (Table 2).

**Table 2:** Scales’ reliability.

Reliability statistics	Cronbach’s alpha	No. of items
Clinical competence	0.968	47
Self‐efficacy scale	0.90	10

There was no significant correlation between demographic data and self‐efficacy and clinical competence. Yet, there was a significant correlation between self‐efficacy and clinical competence (*r* = 0.67, *p* < 0.001).

### 4.2. Self‐Efficacy of Nursing Interns

Table 3 shows the overall self‐efficacy mean score of nursing interns for their reported self‐efficacy during their internship training was 3.33 out of 4 (SD = 0.52). Item 7, “I can remain calm when facing difficulties because I can rely on my coping abilities,” had the highest mean score (*M* = 3.47, SD = 0.70), followed by Item 6, “I can solve most problems if I invest the necessary effort” (*M* = 3.45, SD = 0.65), and Item 9, “If I am in trouble, I can usually think of a solution” (*M* = 3.41, SD = 0.67). Nursing interns reported the lowest mean scores in two items: Item 2, “If someone opposes me, I can find the means and ways to get what I want” (*M* = 3.18, SD = 0.79), and Item 5, “Thanks to my resourcefulness, I know how to handle unforeseen situations” (*M* = 3.12, SD = 0.78).

**Table 3:** Self‐efficacy of nursing interns (*n* = 206).

#	Statements	Mean	SD
1	I can always manage to solve difficult problems if I try hard enough.	3.33	0.64
2	If someone opposes me, I can find the means and ways to get what I want.	3.18	0.79
3	It is easy for me to stick to my aims and accomplish my goals.	3.39	0.73
4	I am confident that I could deal efficiently with unexpected events.	3.25	0.75
5	Thanks to my resourcefulness, I know how to handle unforeseen situations.	3.12	0.78
6	I can solve most problems if I invest the necessary effort.	3.45	0.65
7	I can remain calm when facing difficulties because I can rely on my coping abilities.	3.47	0.70
8	When I am confronted with a problem, I can usually find several solutions.	3.38	0.69
9	If I am in trouble, I can usually think of a solution.	3.41	0.67
10	I can usually handle whatever comes my way.	3.28	0.74

Overall mean		3.33	0.52

### 4.3. Clinical Competence of Nursing Interns

Generally, nursing interns in this study perceived themselves as highly competent during their internship training with an overall mean of 4.42 out of 5 (standard deviation [SD] = 0.56) (Table 4). The average mean for the first dimension, nursing professional behaviors, was 4.52 (SD = 0.70). In this dimension, two clinical competencies, including Item 5, “Adhering to the regulation of patients” and “families’ confidentiality,” and Item 9, “Understanding patient rights,” had the highest mean scores of 4.68 with SD values of 0.60 and 0.65, respectively. The other four clinical competencies with higher mean scores included Item 8, “Maintaining appropriate appearance, attire, and conduct” (mean = 4.65, SD = 0.64), followed by Item 7, “Adhering to ethical and legal standards of practice” (mean = 4.63, SD = 0.67), Item 6 “Demonstrating cultural competence” (mean = 4.62, SD = 0.69), and Item 1, “Following health and safety precautions” (mean = 4.62, SD = 0.69). In addition to this dimension, five clinical competencies had the lowest mean scores, including Item 15, “Communicating verbally with precise and appropriate terminology in a timely manner with healthcare professionals” (mean = 4.39, SD = 0.86), followed by Item 4, “Preventing patients from problem occurrence” (mean = 4.39, SD = 0.81), Item 12, “Applying or accepting constructive criticism” (mean = 4.33, SD = 0.89), Item 11, “Applying appropriate measures and resources to solve problems” (mean = 4.30, SD = 0.85), and Item 13, “Applying critical thinking to patient care” (mean = 4.29, SD = 0.84).

**Table 4:** Clinical competence of nursing interns (*n* = 206).

#	Statements	Mean	SD
1	Following health and safety precautions	4.62	0.68
2	Taking appropriate measures to prevent or minimize risk of injury to self	4.55	0.68
3	Taking appropriate measures to prevent or minimize risk of injury to patients	4.58	0.67
4	Preventing patients from problem occurrence	4.39	0.81
5	Adhering to the regulation of patients’ and families’ confidentiality	4.68	0.60
6	Demonstrating cultural competence	4.62	0.69
7	Adhering to ethical and legal standards of practice	4.63	0.67
8	Maintaining appropriate appearance, attire, and conduct	4.65	0.64
9	Understanding patient rights	4.68	0.65
10	Recognizing and maximizing opportunity for learning	4.56	0.73
11	Applying appropriate measures and resources to solve problems	4.30	0.85
12	Applying or accepting constructive criticism	4.33	0.89
13	Applying critical thinking to patient care	4.29	0.84
14	Communicating verbally with precise and appropriate terminology in a timely manner with patients and families	4.51	0.72
15	Communicating verbally with precise and appropriate terminology in a timely manner with healthcare professionals	4.39	0.86
16	Understanding and supporting group goals	4.46	0.87

*Nursing professional behaviors (average mean)*		4.52	0.70
17	Taking a history for new admissions	4.37	1.02
18	Performing and documenting patient health assessment	4.61	0.70
19	Answering questions for patients or families	4.51	0.69
20	Educating patients or families with disease‐related care knowledge	4.35	0.85
21	Charting and documentation	4.64	0.63
22	Developing care plan for patients	4.28	0.97
23	Performing shift report	4.19	1.09
24	Performing hygiene and daily care routines	4.80	0.51
25	Providing rest and comfort measures	4.71	0.58
26	Assessing nutrition and fluid balance	4.44	0.85
27	Assessing elimination	4.47	0.89
28	Assisting activities and mobility, and changing position	4.67	0.64
29	Providing emotional and psychosocial support	4.51	0.84

*General nursing skills (average mean)*		4.51	0.81
30	Changing intravenous fluid bottle or bag	4.61	0.76
31	Administering intravenous medications (or into the intravenous bag)	4.62	0.75
32	Administering intramuscular medications	4.51	0.98
33	Performing subcutaneous (or intracutaneous) injection	4.78	0.57
34	Administering oral medications	4.85	0.43
35	Performing urinary catheter insertion and care	4.03	1.20
36	Performing sterile techniques	4.57	0.80
37	Performing enema	3.64	1.44
38	Performing upper airway suction	4.33	1.00
39	Performing tracheotomy care	4.17	1.10
40	Performing nasogastric tube feeding and care	4.43	0.94
41	Performing wound dressing care	4.30	1.04

*Core nursing skills (average mean)*		4.40	0.92
42	Performing venipuncture	4.03	1.18
43	Starting intravenous injections	4.30	1.06
44	Administering blood transfusion	3.81	1.23
45	Performing postural drainage and percussion, and oxygen therapy	4.20	1.05
46	Performing pre‐operation and post‐operation care	4.23	1.19
47	Performing chest tube care with underwater seal management	3.73	1.38
Advanced nursing skills (average mean)		4.05	1.15
Overall mean		4.37	0.67

Abbreviation: SD = standard deviation.

The second dimension, general nursing skills, had an average mean of 4.51 (SD = 0.81). Five clinical competencies in the second dimension had the highest mean scores, including Item 24, “Performing hygiene and daily care routines” (mean = 4.80, SD = 0.51), Item 25, “Providing rest and comfort measures” (mean = 4.71, SD = 0.58), Item 28, “Assisting activities and mobility, and changing position” (mean = 4.67, SD = 0.64), Item 21, “Charting and documentation” (mean = 4.64, SD = 0.63), and Item 18, “Performing and documenting patient health assessment” (mean = 4.61, SD = 0.70). Five clinical competencies in this dimension had lowest mean scores, including Item 26, “Assessing nutrition and fluid balance” (mean = 4.44, SD = 0.85), Item 17, “Taking a history for new admissions” (mean = 4.37, SD = 1.02), Item 20, “Educating patients or families with disease‐related care knowledge” (mean = 4.35, SD = 0.85), Item 22, “Developing care plan for patients” (mean = 4.28, SD = 0.97), and Item 23, “Performing shift report” (mean = 4.19, SD = 1.09).

For the third dimension, core nursing skills had an average mean of 4.40 (SD = 0.92). Four clinical competencies in the second dimension had the highest mean scores, including Item 34, “Administering oral medications” (mean = 4.85, SD = 0.43), Item 33, “Performing subcutaneous (or intracutaneous) injection” (mean = 4.78, SD = 0.57), Item 31, “Administering intravenous medications (or into the intravenous bag)” (mean = 4.62, SD = 0.75), and Item 30, “Changing intravenous fluid bottle or bag” (mean = 4.61, SD = 0.76). Meanwhile, three clinical competencies in the third dimension had the lowest mean scores, including Item 39, “Performing tracheotomy care” (mean = 4.17, SD = 1.10), Item 35, “Performing urinary catheter insertion and care” (mean = 4.03, SD = 1.20), and Item 37, “Performing enema” (mean = 3.64, SD = 1.44).

Lastly, the fourth dimension, advanced nursing skills, had an average mean score of 4.05 (SD = 1.15). The clinical competency with the highest mean score was Item 43, “Starting intravenous injections” (mean = 4.30, SD = 1.06), followed by Item 46, “Performing preoperation and postoperation care” (mean = 4.23, SD = 1.19), Item 45, “Performing postural drainage and percussion, and oxygen therapy” (mean = 4.20, SD = 1.05), and Item 42, “Performing venipuncture” (mean = 4.03, SD = 1.18). Two clinical competencies had the lowest mean scores, including Item 44, “Administering blood transfusion” (mean = 3.81, SD = 1.23), and Item 47, “Performing chest tube care with underwater seal management” (mean = 3.73, SD = 1.38).

**Figure 1:**
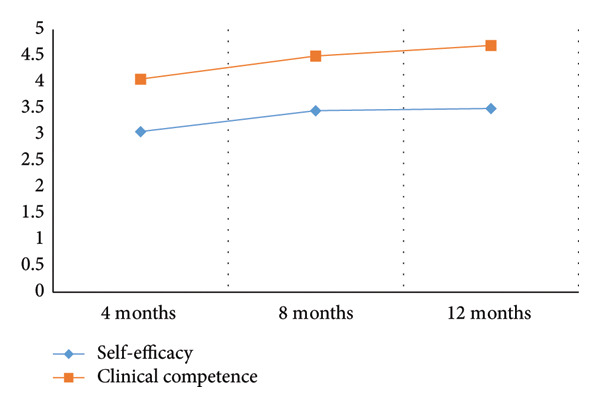
Mean comparisons of self‐efficacy and clinical competence at different stages of internship.

A one‐way ANOVA was conducted to examine between‐group differences in self‐efficacy and clinical competence across three internship durations (4, 8, and 12 months). The results indicated a statistically significant effect of internship duration on both self‐efficacy (*F*(2, 203) = 19.00, *p* < 0.001) and clinical competence (*F*(2, 203) = 38.00, *p* < 0.001) (Table 5). Post hoc comparisons using post hoc Games–Howell revealed that both self‐efficacy and clinical competence scores increased significantly from 4 to 8 months. However, no significant differences were observed between the 8‐ and 12‐month groups (Table 6). These findings reflect between‐group comparisons rather than changes within the same individuals over time (Figure 1). Figure 1 shows the comparison of self‐efficacy and clinical competence scores across internship stages (4, 8, and 12 months).

**Table 5:** ANOVA.

		Sum of squares	df	Mean square	*F*	Sig.
Self‐efficacy	Between groups	9.069	2	4.534	19.800	< 0.001
	Within groups	46.490	203	0.229		
	Total	55.558	205			

Clinical competence	Between groups	17.760	2	8.880	38.035	< 0.001
	Within groups	47.393	203	0.233		
	Total	65.152	205			

**Table 6:** Post hoc Games–Howell.

Dependent variable	(*I*) length	(*J*) length	Mean difference (*I*‐*J*)	Std. error
Self‐efficacy	1.00	2.00	−0.40210^∗^	0.09698
		3.00	−0.44141^∗^	0.07733
	2.00	1.00	0.40210^∗^	0.09698
		3.00	−0.03932	0.08183
	3.00	1.00	0.44141^∗^	0.07733
		2.00	0.03932	0.08183

Clinical competence	1.00	2.00	−0.44653^∗^	0.11430
		3.00	−0.64091^∗^	0.07542
	2.00	1.00	0.44653^∗^	0.11430
		3.00	−0.19438	0.09732
	3.00	1.00	0.64091^∗^	0.07542
		2.00	0.19438	0.09732

^∗^The mean difference is significant at the 0.05 level.

A multiple regression analysis was conducted to examine the extent to which self‐efficacy, duration of internship, social status, GPA, STEP test grade, and having relatives in nursing predicted clinical competence. The overall model was statistically significant (*F*(6, 199) = 40.16, *p* < 0.001, *R* ^2^ = 0.55), indicating that 54.8% of the variance in clinical competence was explained by the model. Self‐efficacy and duration of internship were significant predictors of clinical competence. Other variables, including social status, GPA, STEP test score, and having relatives in nursing, were not significant (Tables 7 and 8).

**Table 7:** Regression coefficients.

Predictor	*B*	SE *B*	*β* (beta)	*t*	*p* value
Self‐efficacy	0.624	0.057	0.576	11.020	< 0.001
Duration of the internship	0.177	0.033	0.285	5.406	< 0.001
Social status	0.016	0.040	0.020	0.400	0.690
Relatives in nursing	0.020	0.058	0.017	0.349	0.728
GPA score	−0.009	0.028	−0.015	−0.307	0.759
STEP test grade	0.003	0.004	0.040	0.795	0.427

**Table 8:** Model summary.

Model	*R*	*R* square	Adjusted *R* square	Std. error of the estimate
1	0.740^a^	0.548	0.534	0.38482

^a^< 0.001.

## 5. Discussion

This study evaluated the nurse intern’s reported self‐efficacy and clinical competence with further examination of the effects of identified predictors such as the length of the internship year to these two variables. As a result, the nursing interns who have participated in this study reported an increased self‐efficacy and clinical competence over the duration of their internship training. A study conducted by Yao et al. [28] suggested that an improved self‐efficacy leads to an enhanced clinical competence. A study on the self‐efficacy and competence of nursing students similarly showed that there was a significant positive correlation between the total score of students’ clinical competence and clinical efficacy, which is highly suggestive of an increase in clinical self‐efficacy, resulting in more clinical competence [15].

The present study conveyed an overall high self‐efficacy among nursing interns in the training program which is a strong reflection of a study by Aboshaiqah et al. [10] using the Arabic version of the Self‐Efficacy for Clinical Evaluation Scale (SECS) which also reported a high level of self‐efficacy by nurse interns in the same country. With the current results, nurse interns have stated high levels of self‐reliance when it comes to finding solutions when facing difficult situations and have stated positively about their coping abilities. Using a similar scale, the findings also showed that nurse interns had higher levels of self‐efficacy (3.33 out of 4) compared to nursing students (average mean score of 2.74) in China [29].

Nursing interns who participated in the present study optimistically perceived themselves as highly competent during their internship year, with an overall mean of 4.42 out of 5. Using the CCQ by Liou and Cheng (2014) with four competency components to evaluate, the nurse interns have rated general nursing skills with the highest rating of 4.51 as its average mean, nursing professional behaviors is the second with an average mean of 4.52, third is the core nursing skills with 4.40, and the advanced nursing skills is the lowest with 4.05. The study revealed that the amount of clinical training received enabled the nurse interns to report high competency in basic nursing skills and moderate efficacy when it comes to advanced nursing skills which require years of practice and expertise to attain. These results support another current study among nursing interns in the Al Jouf Region in KSA which showed that the majority of nurse interns displayed moderate readiness which prompts further development and support in different areas of their clinical training [13]. Both findings of these studies are consistent with the expected transition from a novice to an advanced beginner included in Dr. Patricia Benner’s Five Stages of Clinical Competence [30]. In addition, the present study’s mean scores of nursing interns in the four dimensions ranged from 4.05 to 4.51 which are higher compared to the previous study among nursing students (mean scores range: 3.98–4.13) in Saudi Arabia [31]. Other studies also supported the findings of the current study including among novice nursing students in Taiwan [32], graduate nurses in Australia [33], and nurse practitioner students in Norway [34].

The absolute impact of self‐efficacy on the clinical competence of nurse interns is evident in the results of this study. The exhibited high levels of self‐efficacy of nurse interns have a direct relation to their clinical competence; high self‐efficacy leads to enhanced clinical competence. This finding is consistent with a previous study among a cross‐sectional sample of 887 Chinese nursing students in the internship training period of their education program where self‐efficacy had a positive impact on their clinical competence [28]. Similarly, self‐efficacy was also found to be a mediating factor between professional identity and clinical competence [28].

Aside from self‐efficacy, there are other factors that affect the clinical competence of nursing interns in the current study, including having relatives/friends studying/working in nursing and the duration of the internship program. These findings are comparable with other previous studies; for example, clinical learning environment and professional interest were found to predict clinical competence among Chinese nursing students [35]. Specifically, during the COVID‐19 pandemic, adaptive coping strategies significantly affected the clinical competence among South Korean nursing students [36]. In Saudi Arabia, gender and year of education (i.e., fourth year and internship year) were reported as predictors of the perceived clinical competence of nursing students [3]. In another study among Saudi nursing students, age, gender, marital status, and course were indicated as predictors of their clinical competence [31].

The overall effectiveness of the internship program has been firmly established due to a wide range of research conducted regarding its utilization. Nursing interns perceived the preceptorship program as effectively contributing to their role transition in becoming professional nurses [12]. Likewise, programs designed specifically for interns were a critical factor affecting the participants’ ability to gain the required knowledge and a significant factor that facilitated their clinical practice [37]. One important breakthrough regarding internship programs that was verified in this current study is that nursing interns who have been in the program up to 8 months had better clinical competence than those who have spent 1–4 months of clinical training. This finding implied that clinical competence was enhanced with continuous increase of time during the gradual intensification of the internship training period. This finding is consistent with a previous study in China where the development of professional competence increased from the end of the second month to the end of the sixth month of the clinical practice period among undergraduate nursing students [38]. In addition, another significant outcome of this study is the substantial increase in self‐efficacy and clinical competence on the first two phases of the internship program, 4‐ and 8‐month clinical training, respectively; however, no significant increase was observed on the last phase of the program lasting until the 12th month. The findings are comparable to those of Missen et al. [39], who stated that implementing supportive transition programs, irrespective of the duration, assists with positive job satisfaction levels and increased confidence and retention rates of newly graduated nurses.

Moreover, with the findings of the current study, a challenge was posed to nurse educators and academic institutions in KSA to further enhance and reconsider certain aspects of the nursing curriculum. As the world continuously shifts to a more advanced setting, the use of competency‐based education curriculum which places emphasis on practical learning to promote learner engagement is highly beneficial in enhancing students’ clinical competency [23]. Indisputably, the internship program is proven highly effective in enhancing students’ clinical competence [3]. However, in curriculum development, the content and the length of delivery are not the sole priorities, but it also involves how to achieve the program outcomes while considering the stakeholders’ expectations [23].

## 6. Study Limitations

There are several limitations that are worth noting in the present study. One notable limitation of this study is the use of convenience sampling, which may introduce selection bias and limit the generalizability of the findings. Since participants were not randomly selected, the sample may not fully represent the broader population, potentially affecting the external validity of the results. Future studies should consider employing probabilistic sampling methods to enhance representativeness and improve the robustness of the findings. Further, the data of this study were collected in one setting only, and the use of self‐reported tools restricted the generalizability of the study findings. Third, this study used a cross‐sectional design which made it difficult to explore the assessment of causality or changes over time compared to longitudinal studies. As such, the researchers recommended complementing the study results with another quantitative study with a larger sample, to be conducted using a longitudinal approach, and with qualitative studies. The last potential limitation of this study could be the reliance on self‐reported measures of competence, which may be subject to social desirability bias. Participants might have overestimated their competence levels to align with perceived expectations, potentially affecting the accuracy of the findings.

### 6.1. Nursing Implications

This study demonstrates that nursing interns show significant increases in self‐efficacy and clinical competence between 4 and 8 months of internship, with no significant improvements noted from 8 to 12 months. These findings suggest that the early and middle phases of the internship are the most critical for clinical skill development and confidence‐building. Therefore, clinical educators and preceptors should prioritize intensive training, feedback, and mentorship during this formative period.

The observed level in nursing competence after 8 months may reflect limitations in the clinical learning environment rather than a true ceiling of skill acquisition as without further growth, this period may represent an inefficient use of resources and time for both interns and institutions. During the early months of clinical exposure, newly graduated nurses often experience rapid development as they apply foundational knowledge and adapt to real‐world practice. However, as they become more familiar with routine tasks, opportunities for new learning may decline. Repetition of similar responsibilities and limited rotation across specialties can contribute to lack of progress in professional growth. These insights highlight the need for dynamic training models and mentorship programs that sustain competence growth beyond the initial transition period.

## 7. Conclusion and Recommendation

This study highlights the strong link between self‐efficacy and clinical competence among nurse interns, with both showing significant progress up to the 8‐month mark. However, no added benefit was seen with longer internships. These findings indicate that the most critical period for skill acquisition and confidence building occurs within the first 8 months of clinical training.

Given the ongoing global nursing shortage and the urgent need to support and strengthen the nursing workforce, these results suggest a potential opportunity to reassess the current length of internship programs. By concentrating training efforts and resources in the early to mid‐phase—when the most growth occurs—it may be feasible to shorten internship programs to 8 months without compromising educational or clinical outcomes.

Shortening the internship duration could accelerate the transition of competent and confident nurses into the workforce, thereby helping to address staffing gaps more efficiently. In addition, healthcare institutions and nursing education providers should consider redesigning internship curricula to maximize learning and competency development in the early months through structured mentorship, simulation‐based training, and focused clinical rotations.

It is also recommended that evaluation mechanisms be strengthened to monitor intern progress throughout the program, ensuring that those who meet competency benchmarks can safely and confidently transition into practice. Flexibility in program duration, based on demonstrated competence rather than fixed timeframes, may further enhance efficiency and workforce responsiveness. This adjustment may also help ease nursing shortages by accelerating workforce entry and allowing institutions to reinvest saved resources into targeted support, such as mentorship and professional development, to boost early‐career success and retention.

Further research and policy discussions are warranted to explore the feasibility and long‐term impacts of such changes on nurse readiness, retention, job satisfaction, and patient care quality. Longitudinal research is recommended to track the sustained effects of shortened internship programs on clinical performance and professional development over time. Lastly, future research employing longitudinal designs is recommended to better understand the directionality and causal pathways of these relationships.

## Conflicts of Interest

The author declares no conflicts of interest.

## Funding

This research received no grant from any funding agency in the public, commercial, or not‐for‐profit sectors.

## Data Availability

The data that support the findings of this study are available on request from the corresponding author. The data are not publicly available due to privacy or ethical restrictions.
